# Sense of Coherence: factorial structure and association with oral health - a study of Norwegian adults

**DOI:** 10.2340/aos.v84.45272

**Published:** 2025-12-30

**Authors:** Victoria Xenaki, Anne Nordrehaug Åstrøm

**Affiliations:** a Department of Clinical Dentistry, Faculty of Medicine, University of Bergen, Bergen, Norway; b Oral Health Centre of Expertise in Western Norway, Bergen, Norway

**Keywords:** Sense of coherence, oral health, psychometric properties

## Abstract

**Objective:**

Assess the construct validity of Sense of Coherence (SOC) by testing a one- and a three-factor structure and associations with oral health indicators.

**Method:**

In all, 9000 adults were randomly selected from a national population panel of 83,000 and 1,557 completed telephone interviews. SOC was measured using the 13-item Norwegian version of the original 29-item Orientation to Life questionnaire. Oral health was assessed as oral impacts on daily performances (OIDP), periodontal symptoms and attitudes towards oral health. Descriptive statistics and multivariable logistic regression analyses were conducted using Statistical Package for the Social Sciences (SPSS). Confirmatory factor analysis (CFA) was performed using the Lavaan package in R.

**Results:**

Confirmatory factor analysis provided satisfactory fit to a modified one-factor model; comparative fit index (CFI) = 0.940, root mean squared error of approximation (RMSEA) = 0.062, Tucker-Lewis index (TLI) = 0.925. Factor loadings ranged from 0.360 to 0.798. Metric invariance was obtained across sexes. Adults having strong SOC were less likely to report oral impacts and periodontal symptoms, and more likely to report positive oral attitudes.

**Conclusion:**

This study demonstrated construct validity of the Norwegian version of SOC 13 and that, in the presence of socio-demographic factors, SOC is an important contributor to oral health outcomes. SOC should be considered in oral health educational and promotional interventions among Norwegian adults.

## Introduction

Sense of Coherence (SOC) is the main psychosocial construct of the Salutogenesis theory and refers to the origin of health rather than that of diseases [1]. SOC describes a health protective life orientation with three interrelated components, *comprehensibility –* the way people make sense of the world, *manageability –* how they use resources to respond to it, and *meaningfulness –* how they feel that these responses are meaningful and emotionally sensible [1, 2]. Identifying why people achieve and maintain good health in adverse situations and cope with income related health adversity indicates what might regulate health [3]. A strong SOC has been suggested to facilitate successful coping with stressors and thus maintenance and improvement of health and oral health [4]. This makes SOC an important aspect to consider when designing health and oral health promoting interventions [4].

To measure SOC, a scale exists in two main forms; the original version of the SOC scale consists of 29 items and the abbreviated version covers between 3 and 13 items [4]. Whereas psychometric properties of both forms, in terms of reliability and validity, have been found to be satisfactory across cultural contexts, there is less evidence of whether this instrument should be interpreted as one-dimensional or alternatively a multidimensional construct. Although the abbreviated SOC-13 instrument has been described in terms of three correlated components — manageability, comprehensibility, and meaningfulness, few studies have validated this factor structure in adult populations and even fewer have assessed whether the factor structure is equivalent across population subgroups [5]. Population based studies have supported a simple one-factor structure of various abbreviated versions of the SOC scale that does not differentiate clearly between SOC components [6–8]. Other studies have provided support for a multifactorial structure, however with high correlations found between three SOC factors thus limiting their use as separate indicators [9–10].

A number of studies have shown that strong SOC associates with better oral health outcomes such as clinical- and self-reported oral health [11–17]. Thus, cross-sectional and longitudinal studies have reported on a negative relationship between SOC and dental caries, gingival/periodontal problems, malocclusion, oral mucosal lesions, number of teeth, and problems related to use of protheses [11–17]. In a study of adults in northern Norway, stronger SOC was associated with more frequent dental visits and in turn with more serious periodontitis [17]. In contrast, Cyrano et al. [18] did not support a significant association between SOC and clinical periodontal outcomes among Brazilian adults, but revealed that stronger SOC predicted less frequent self-reported gingival bleeding. No association between baseline SOC and change in number of teeth with periodontal pocketing was reported from a study of the Finnish adult population [15]. Several studies, among them a recent review, have revealed that more favourable oral health related behaviours were observed among individuals with stronger SOC, suggesting SOC to be an important psychosocial determinant of oral health [12, 19]. Some studies have investigated whether SOC interacts with socio-demographic characteristics and plays a moderating and or mediating role with respect to inequality in health and oral health status [20, 21].

Available evidence of the influence of SOC on clinical- and self-reported oral health indicators is still conflicting [11–19]. Possible reasons for inconsistent findings are the influence of cultural differences, use of different abbreviated versions of the SOC instrument, and other methodological caveats. Some studies are based on convenience samples, thereby precluding generalisation to whole populations. Others are restricted to one particular sex, patient group, or study setting. Moreover, the association of SOC with oral health indicators have not been adjusted properly for potential confounding variables in many studies. Further population-based studies are therefore needed; it has also been requested to get more information about how SOC interact with socio-demographic factors and oral health related behaviours.

This study takes a step further to understand the utility of SOC among Norwegian adults using a 13-item Norwegian version of SOC [17]. Focusing a national sample of 16–79-year olds, this study aimed to address the question of construct validity of a 13-item SOC instrument by testing two alternative factor structures, a one- and a three-factor structure, and to investigate how SOC is associated with oral health outcomes after adjustment for socio-demographic factors. It was hypothesised that responses to the 13-item Norwegian version of SOC would be explained better by one than three underlying factors, and that the 13 items would show an equivalent factorial and factor loading structure across sexes. It was further hypothesised that stronger SOC would be associated with better oral health indicators after adjustment for socio-demographic factors.

## Material and methods

The present study used data from a telephone based oral health survey conducted in Norway in 2018. A random sample of 9,000 Norwegian adults aged 16–79 years was recruited by probability proportional to size of region and age groups from a national population panel of 83,000 Norwegian adults, administered by NORSTAT (www.nortst.no). Participants were recruited with replacement until the pre-calculated sample size of respondents was reached. The sample was weighted on age and sex according to official population statistics. Participation in the survey was voluntary and anonymous. Ethical permission was applied for at the Regional Committees for Medical and Health Research Ethics (REC). NORSTAT has its own privacy representative ensuring that the rules of the privacy act are followed. NORSTAT was responsible for sample selection, pre-calculation of the sample size, administration of the telephone interview, and preparation of anonymous data files. All respondents who completed the telephone based oral health survey were included in the present study (*n* = 1,557).

### Exposure variable

SOC was assessed (with permission through personal communication with Avishai Antonovisky) by telephone interviews, using the abbreviated 13-item Norwegian version of the original 29-item Orientation to Life questionnaire [17, 19]. Participants responded to the following questions/statements: *‘Do you feel that you really do not care about what happened around you’?, ‘Has it happened in the past that you were surprised of the behaviour of people whom you thought you knew well’, ‘Has it happened that you were disappointed of persons that you trusted’*, ‘*Until now has your life had no clear intentions – clear intentions’*, ‘*Do you feel that you are unfairly treated’*, ‘*How often do you feel to be in an uncommon situation that you do not know how to cope with’*, ‘*To do daily activities are a source of joy and satisfaction – a source of pain and are boring’*, ‘*Do you have quite contradicting feelings and thoughts?’, ‘Does it happen that you have feelings that you do not want to have?’, ‘How often have you felt to be a looser? ’*, *‘When something has happened have you often experienced that you overestimated or underestimated the meaning of it – you evaluated it correctly*’, *‘How often do you feel that things you are doing are less meaningful?’, ‘How often do you have feelings that you are not sure you can control’*. Replies were given on a 7-point Likert scale ranging from 1 = very often/always/considered correct to 7 = seldom or never/never/considered incorrect. Sum score was created by adding the single items whereby negatively worded items were reversed.

### Socio-demographic covariates

Covariates included age, sex, educational level (participants reported the number of years of their full time education), marital status, and place of birth (inside/outside Norway). Household income (monthly per consumption unit in Norway NOK) was obtained from the tax authorities

### Oral health outcomes

Five indicators of oral health outcomes were utilised in terms of oral impacts on daily performance (OIDP) [22], self-reported periodontal symptoms, oral health attitudes, and dental attendance. *The OIDP* was assessed using the Norwegian version of the eight item OIDP inventory, previously shown to be reliable and valid when applied in the Norwegian adults population 16–79 years of age [23]. The OIDP frequency index refers to difficulty carrying out eight daily life activities *‘During the past 6 months, how often have problems with your mouth or teeth caused you any difficulty with: eating and enjoying food, speaking and pronouncing clearly, cleaning teeth, sleeping and relaxing, smiling and laughing, emotional status, socialisation and contact with people’.* The original responses to each item were (0) never, (1) once or more a month, (2) once or more a week, and (3) every day/nearly every day. For statistical analyses, the frequency items were dichotomised as 0 not affected (comprising of original responses 0) and 1 affected (comprising of original responses 1, 2 and 3). The cut off point was justified by the frequency distribution of the eight OIDP items. An OIDP sum count (SC) score (range 0–8) was constructed by summing the dichotomised frequency items. This SC score was subsequently dichotomised into 0 (no impacts) and 1 (at least one impact) in accordance with what is recommended in the literature. The internal consistency reliability (Cronbach’s alpha based on standardised items) for the OIDP SC score was 0.82.

Periodontal symptoms were assessed by four single items. *‘How often does your gingiva bleed during interdental cleaning’*, (1) every time to (4) never, *’Have you had problems with your gums the previous month’* with responses (1) yes, (0) no. *‘Have the dentist told you that you have periodontal problems’* with responses (1) yes and (0) no. *‘Have you got treatment for periodontal disease’* with responses (1) yes, (0) no. A sum score periodontal problems was constructed and dichotomised into 1 = at least one periodontal problem confirmed and 0 = no periodontal problems confirmed.

Attitudes towards oral health was measured by six items in terms *of ‘I take care of my teeth on daily basis, I get the dental care that I need, I need dental care but postpone, I do not do my oral hygiene properly, I control my snacking, and I consider my oral health as less important’.* Each item was evaluated on a 5-point Likert scale ranging from 1 = completely agree to 5 = completely disagree. A sum score was constructed, after reversing the scores of the positively worded items. This sum score was dichotomised on a median split into 0 = negative oral attitude and 1 = positive oral attitude. Cronbach’s alpha for the sum scale was 0.72*.*

### Statistical analyses

**Figure 1:**
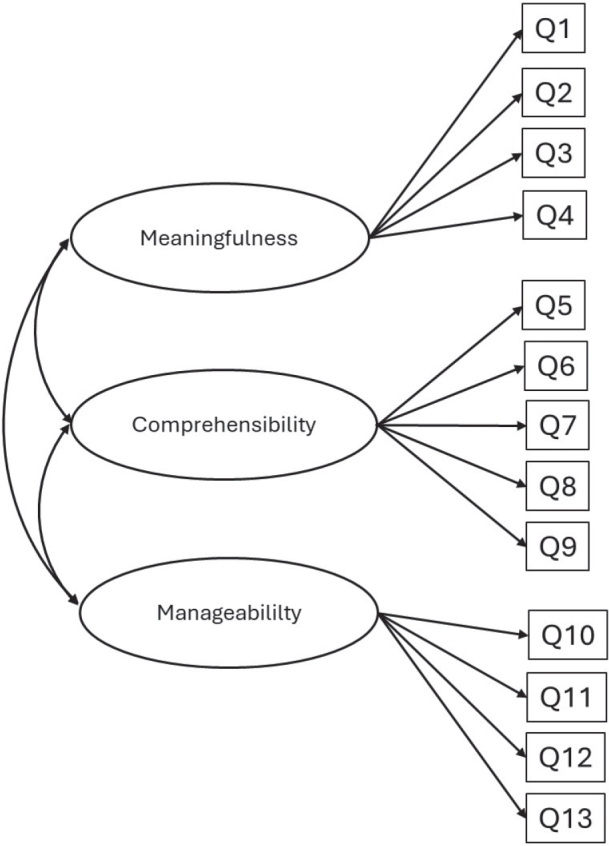
The hypothesised three factor model. Meaningfulness: Q1: Don’t care about surroundings, Q2: Until now lack of goals and meaning. Q3: To do daily activities, a source of pain and boredom, Q4: Lack of meaning in daily activities. Comprehensibility: Q5: Surprised regarding well known persons, Q6: Do not know what to do in uncommon situations, Q7: Confusing thoughts and feelings, Q8: Unwanted feelings, Q9: How events are considered. Manageability: Q10: Disappointed about trusting others, Q11: Feel treated unfair, Q12: Feeling as a looser. Q13: Lack of control.

**Figure 2:**
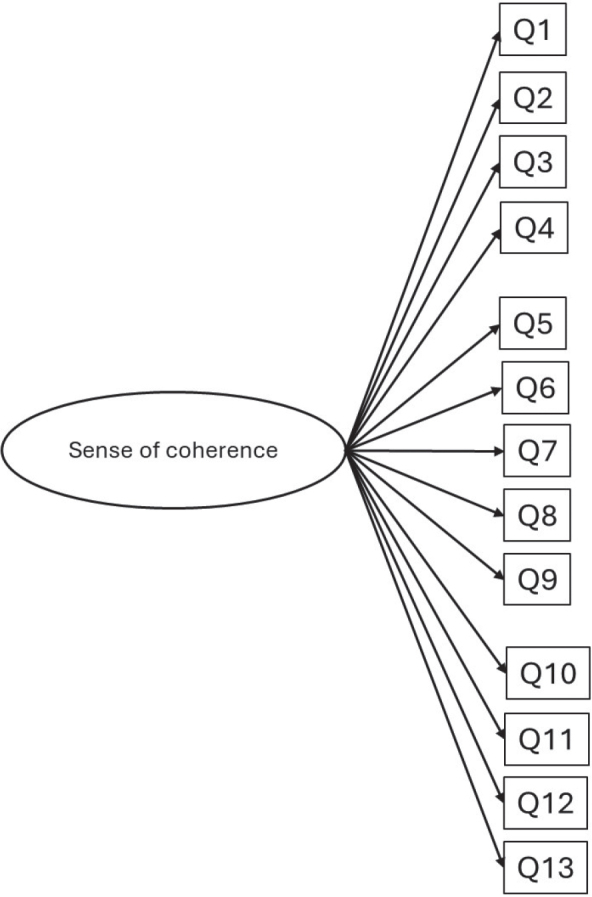
The hypothesised one factor model.

Descriptive statistics, cross-tabulations, chi-square statistics, and multivariable logistic regression analyses, with dichotomy outcome variables, were conducted using Statistical Package for the Social Sciences (SPSS), version 25.0 (IBM Corp., Armonk, NY, USA). Confirmatory factor analysis (CFA) was performed using the Lavaan package in R (R Core Team 2020. R: A language and environment for statistical computing. R Foundation for Statistical Computing, Vienna, Austria) [24–26]. Confirmatory factor analysis was performed to test whether the Norwegian data were consistent with an *a priori* hypothesised three-factor model (Figure 1) of SOC including three latent variables in terms of comprehensibility (with five items assigned), manageability (with four items assigned), and meaningfulness (with four items assigned). This 3-factor model was compared with an alternative *a priori* hypothesised one-factor model (unidimensional) (Figure 2), including one latent factor underlying all 13 items in the abbreviated SOC scale. The adequacy of overall model fit was estimated using chi-square test statistics and the following supplemental fit indices: root mean squared error of approximation (RMSEA), the comparative fit index (CFI), and the Tucker-Lewis index (TLI). A non-significant chi-square test indicates that the model is a plausible representation of the observed variables. Due to the high sensitivity of the chi-square statistics to the sample size, additional fit indices were used. Moreover, in line with conventional recommendations of Hu and Bentler [26], a good model fit was indicated by RMSEA less or equal to 0.06 and a CFI greater or equal to 0.90. Potential sources of misfit were examined with the help of modification indices, which gave basis for the re-specification of the measurement model. Multigroup analyses were performed with CFA to test whether the best fitting model was invariant across gender. Configural invariance (equal forms) was tested by fitting the final measurement model across gender and supported if the model had a satisfactory fit (based on above-mentioned fit indices). Metric invariance (equal factor loadings) was tested by constraining factor loadings in all groups and by comparing the constrained model with the baseline model (configural invariance model) in which factor loadings were free to vary. Metric invariance was supported if Chi-square delta was non-significant and CFI delta was less than 0.002. As item responses were assessed using ordinal scales, weighted least squares method was used to estimate model parameters. For multiple variable regression analyses, two models were built for each oral health outcome, whereby socio-demographics were entered in Model I, III, and V followed by SOC entered as an additional variable in model II, IV, and VI.

## Results

### Sample profile

A total of 1,557 participants constituted the final sample (804 men and 753 women). The mean age was 44.0 years (17.5), range 16–79 years. Totals of 27.9%, 49.7%, and 22.4% were respectively of younger (16–30 years), middle (31–43 years) and older (44–79 years) ages. The sex distribution in the sample was 51.6% males and 48.4% females. Corresponding figures in the 2018 Norwegian adult population was 50.4% and 49.6%. Among the respondents, a total of 92.0% were native Norwegians and 15.1% reported university education above 4 years as their highest level. Corresponding figures in the 2018 Norwegian population were 88.5% and 10.3% (SSB population characteristics 2018). The similarity in sociodemographic distribution between the sample and its population provides some support to the external validity of the estimates.

### Factor structure of Sense of coherence

Table 1S (Supplementary) shows the descriptive statistics in terms of mean and standard deviation (SD) for each of the 13 items included in the abbreviated SOC scale. Mean values ranged from 4.0 (‘disappointed about trusted others’) to 5.2 (‘don’t care about surroundings’) and Cronbach’s alpha amounted to 0.88.

Table 1 depicts the goodness of fit statistics for the two hypothesised factor structures of the 13-item Norwegian SOC scale. The initially proposed one-factor (Chi-square = 889.95, CFI = 0.872, RMSEA = 0.101, TLI = 0.847) and three-factor (Chi square 748.790, CFI = 0.890, RMSEA = 0.094, TLI = 0.861) models were not an acceptable fit to the data according to any of the fit indices employed. Inspection of modification indices suggested that estimating freely the correlations between the random errors of one item pair (‘Has it happened in the past that you were surprised by the behaviour of people you thought you knew well’ and ‘Has it happened that people you trusted have disappointed you’) would improve the fit of the 3-factor model. After re-estimating, the above suggested modifications improved the fit indices of the 3-factor model (chi-square = 461.516, CFI = 0.951, RMESA = 0.065, TLI = 0.937). The standardised factor loadings ranged from 0.409 to 0.813, and were all statistically significant in the expected direction (*p* < 0.001). The covariances of the three latent variables (inter factor correlations) approached or exceeded the cut-off point of 0.850 being in the range of 0.818–1.027. This indicates lack of discriminative validity of the three factors and a high degree of collinearity in the data.

**Table 1:** Overall goodness of fit indices (maximum likelihood estimation) for 1- and 3-factor models of Sense of Coherence.

All	Chi square test (df)	CFI	RMSEA	TLI
Initially hypothesised 1-factor model	889.95 (65)**	0.872	0.101	0.847
^a^1-factor model – modified	438.37 (63)**	0.940	0.062	0.925
Initially hypothesised 3-factor model	748.790 (62)**	0.890	0.094	0.861
^b^3-factor – modified	461.516 (61)**	0.951	0.065	0.937
Modified 1-factor model – men only	243.895 (63)**	0.942	0.061	0.044
Modified 1-factor model – women only	240.370 (63)**	0.932	0.067	0.932

CFI: comparative fit index; df, degrees of freedom; RMSEA: root mean squared error of approximation; TLI: Tucker-Lewis index.

a With modification indices.

b with modification indices.

** *p* < 0.001.

Modification indices of the 1-factor model was suggested to estimate freely the random errors of two item pairs (‘Don’t care about surroundings’ and ‘Disappointed about trusted ones’) and (‘Do you have very confusing thought and feelings’ and ‘Does it happens that you have feelings that you do not want to have’). Re-estimation provided satisfactory fit of the modified 1-factor model (chi square = 438.37, CFI = 0.940, RMSEA = 0.062, TLI = 0.925). All factor loadings were statistically significant (*p* < 0.001) and loaded in the expected direction on the SOC latent factor, ranging from 0.360 to 0.798 (Table 2S).

Using multigroup CFA, the modified 1-factor model was tested for equivalence in factor structure (configural invariance) and factor loading (metric invariance) across sexes. The fit indices for men and women separately were satisfactorily (Men: chi-square = 243.895 degrees of freedom [df] = 63, CFI = 0.942, TLI = 0.044, RMSEA = 0.061, Women: chi square = 240.370 df = 63, CFI = 0.932, TLI = 0.932, RMSEA = 0.061). Moreover, the fit indices for the combined data (men and women) in the first unconstrained baseline model were acceptable (chi square = 484,325, CFI = 0.945, RMSEA = 0.067, TLI = 0.932), thus providing support for configural invariance; indicating that the number of factors and patterns of indicator-factor loadings were identical across men and women. Comparing the unconstrained baseline model (CFI = 0.945) with the constrained model (CFI = 0.946) provided delta CFI = 0.001 which is below the value of 0.002, and thus indicates metric invariance or equal factor loadings across sexes.

### Sense of coherence and oral health outcomes

According to the one factor solution, a sum score of all 13 items were constructed with mean 55.8 (SD = 12.12) ranging from 15 to 82. The lower the score on this sum scale, the weaker the SOC. For analyses a three-level categorical variable was constructed in terms of 1 = weaker SOC (original scores 15–49), 2 = average SOC (original scores 50–59), and 3 = stronger SOC (original scores 60–82). Totals of 30.1%, 29.2%, and 47% presented with respectively, weaker, averaged, and stronger SOC.

In the total sample, 37.6% reported experience with periodontitis symptoms, 49.1% positive attitudes, 55.7% annual dental attendance, and 37.3% at least one oral impact on daily performances (OIDP > 0). As shown in Table 2, the highest proportions reporting at least one oral impact and periodontal symptoms were observed among the youngest group of participants, participants with the lowest annual household income, those born outside Norway, and among those with lower level of education. Highest proportion of participants with positive oral attitudes were found among women, the older age group, those with the highest household income, Norwegian born participants, married people, and those with the highest level of own education. Annual dental attendance varied with all socio-demographics except marital status and own educational level. A larger proportion of participants with weak than with strong SOC reported oral impacts and periodontal symptoms. A larger proportion of participants with strong than with weak SOC reported positive oral attitudes and annual dental attendance.

**Table 2:** Sample distribution of Norwegian adults by socio-demographics, Sense of Coherence, and oral health outcomes.

Variables	% (*n*)	% (*n*) OIDP > 0	% (*n*) Perio symptoms yes	Attitudes-positive % (*n*)
**Sex**				
Men	51.6 (804)	37.6 (302)	36.7 (295)	45.5 (366)
Women	48.4 (753)	37.1 (279)	38.5 (290)	53.0 (399)*
**Age groups**				
Younger (16–30 years)	27.9 (435)	46.0 (200)	41.6 (181)	39.5 (172)
Middle (31–43 years)	49.7 (774)	36.7 (284)	37.3 (289)	53.7 (416)
Older (44–79 years)	22.4 (348)	27.9 (97)	33.0 (115)*	80.5 (280)**
**Household income (annually)**				
0–500.000 NOK	28.2 (337)	41.8 (141)	43.6 (147)	42.1 (142)
500.001–100,000 NOK	45.3 (542)	36.9 (200)	36.0 (195)	52.2 (283)
>1,000,001 NOK	26.5 (317)	31.2 (99)*	35.3 (112)*	56.5 (179)**
**Place of birth**				
Norway	91.9 (1,431)	36.2 (518)	36.8 (526)	50.1 (717)
Outside Norway	8.1 (126)	50.0 (63)*	46.8 (59)*	38.1 (48)*
**Marital status**				
Single	30.7 (471)	36.9 (174)	40.6 (191)	45.6 (215)
Married	69.3(1,065)	37.6 (400)	36.4 (388)	51.2 (545)*
**Own education**				
Undergraduate	40.8 (634)	41.5 (263)	40.2 (255)	43.8 (278)
University up to 3 years	27.8 (431)	36.4 (157)	36.9 (159)	50.1 (216)
University above 3 years	31.4 (488)	32.2(142)*	33.8 (149)	56.5 (249)**
**(SOC)**				
Weak	30.1 (469)	52.9 (248)	47.5 (223)	29.2 (137)
Averaged	29.2 (455)	38.2 (174)	40.4 (184)	45.9 (209)
Strong	40.7 (633)	25.1 (159)**	28.1 (178)**	66.2 (419)**

OIDP: oral impacts on daily performances; SOC, sense of coherence.

Oral health outcomes % (*n*) by socio-demographics and sense of coherence.

** *p* < 0.001,

* *p* < 0.05.

As shown in Table 3, larger proportions of men than women (43.8% vs. 37.3%), older than younger participants (65.2% vs. 19.5%), and larger proportions of participants with higher than lower household income (54.9% vs. 30.6%) and higher than lower education (50.6% vs. 33.0%) reported strong SOC.

**Table 3:** Sense of coherence according to socio-demographic characteristics.

	Strong SOC % (*n*)
**Sex**	
Men	43.8 (352)
Women	37.3 (281)*
**Age groups**	
Younger	19.5 (85)
Middle aged	41.5 (3,219
Older	65.2 (227)**
**Household income**	
0–500.000	30.6 (103)
500.001–100,000	41.7 (226)
>100,000	54.9 (174)**
**Place of birth**	
Norway	41.0 (587)
Outside Norway	36.5 (46)
**Own education**	
Undergraduate	33.0 (209)
University up to 3 years	40.6 (175)
University above 3 years	50.6 (247)**
**Marital status**	
Single	32.3 (152)
Married	44.5 (474)**

SOC: sense of coherence.

** *p* < 0.001,

* *p* < 0.05.

As shown in Table 4, the independent association of SOC with oral health outcomes was assessed in sequential logistic regression models. When OIDP was regressed on socio-demographics (Model I), age, household income, and place of birth occurred as significant covariates. Compared to the younger participants, middle aged and older participants were odds ratio (OR): 0.7 (95% confidence interval [CI]: 0.5–0.9) and OR: 0.4 (95% CI: 0.3–0.6) times less likely, whereas participants from outside Norway were about two times more likely to report oral impacts. When entering SOC in Model II, only place of birth remained statistically significantly associated with OIDP. Compared with participants having weaker SOC, those with average and strong SOC were respectively, 0.5 (95% CI: 0.4–0.7) times and 0.3 (95% CI: 0.2–0.4) times less likely to report oral impacts. In Model III, place of birth and age were significant covariates of periodontal symptoms. After including SOC in Model IV, the associations with age and place of birth did not remain significant. The final model revealed that compared to those with weaker SOC, participants with average and stronger SOC were respectively, OR: 0.7 (95% CI: 0.5–1.0) and OR: 0.5 (95% CI: 0.4–0.7) times less likely to report periodontal symptoms. Significant associations between age and positive oral attitudes and between household income and positive attitudes (Model V) remained statistically significant after entering SOC (Model VI). Compared to those with a weaker SOC, participants with average and stronger SOC were respectively OR: 2.0 (95% CI: 1.5–2.7) and OR: 3.5 (95% CI: 2.5–4.9) times more likely to present with positive oral health attitudes. Two-way interactions between SOC and socio-demographic variables on each oral health outcome were not statistically significant.

**Table 4:** Oral impacts on daily performances and periodontal symptoms regressed on socio-demographic factors and Sene of Coherence.

	OIDP > 0 Model I	OIDP > 0 Model II	Periodontal symptoms Model III	Periodontal symptoms Model IV	Positive oral attitudes Model VII	Positive oral attitudes Model VIII
	OR (95% CI)	OR (95% CI)	OR (95% CI)	OR (95% CI)	OR (95% CI)	OR (95% CI)
Socio-demographic						
Age						
Younger	1	1	1	1	1	1
Middle	0.7 (0.5–0.9)	0.8 (0.6–1.1)	0.8 (0.6–1.1)	0.9 (0.6–1.3)	1.6 (1.2–2.2)	1.4 (0.9–1.8)
Older	0.4 (0.3–0.6)	0.7 (0.5–1.1)	0.6 (0.4–0.9)	0.9 (0.6–1.3)	3.9 (2.7–5.6)	2.5 (1.7–3.7)
Household income						
0–500.000 NOK	1	1	1	1	1	1
500,001–1,000,000	0.7 (0.5–1.1)	0.8 (0.5–1.2)	0.7 (0.5–1.0)	0.7 (0.5–1.1)	1.5 (1.1–1.9)	1.3 (0.9–1.9)
>1,000,000	0.5 (0.3–0.8)	0.7 (0.4–1.1)	0.7 (0.5–1.1)	0.8 (0.5–1.3)	1.8 (1.2–2.7)	1.5 (1.0–2.3)
Civil status						
Single	1	1	1	1	1	1
Married	1.3 (0.9–1.8)	1.4 (1.0–1.9)	0.9 (0.7–1.4)	1.1 (0.7–1.4)	0.8 (0.6–1.2)	0.8 (0.5–1.1)
Education						
Undergraduate	1	1	1	1	1	1
University up to 3 years	0.9 (0.7–1.3)	1.0 (0.7–1.4)	0.9 (0.7–1.3)	0.9 (0.7–1.3)	0.9 (0.7–1.3)	0.9 (0.7–1.3)
University above 3 years	0.8 (0.6–1.1)	0.8 (0.7–1.2)	0.8 (0.6–1.1)	0.8 (0.6–1.2)	1.2 (0.9–1.6)	1.1 (0.8–1.5)
Place of birth						
Norway	1	1	1	1	1	1
Outside Norway	1.9 (1.2–2.9)	1.8 (1.2–2.9)	1.6 (1.0–2.4)	1.5 (0.9–2.4)	0.6 (0.4–0.9)	0.6 (0.4–0.9)
Sense of coherence						
Low SOC		1		1		1
Average SOC		0.5 (0.4–0.7)		0.7 (0.5–1.0)		2.0 (1.5–2.7)
High SOC		0.3 (0.2–0.4)		0.5 (0.4–0.7)		3.5 (2.5–4.9)

OIDP: oral impacts on daily performances; SOC: sense of coherence; OR: odds ratio; CI: confidence interval.

Logistic regression, OR and 95% CI.

## Discussion

This study assessed the factorial structure of the13-item SOC scale focusing on a representative sample of Norwegian adults aged 16–79 years. The findings provide further support to the construct validity of SOC by indicating that a modified version of a 1-factor model fitted the data more satisfactorily than a modified 3-factor model and thus, provided the most parsimonious characterisation of the SOC construct. Moreover, the unidimensional SOC construct was invariant across sexes with respect to factor structure and factor loadings. Robust associations in the expected direction were observed between SOC and various oral health indicators after adjustment for socio-demographic factors, known to be associated with oral health outcomes and SOC. This appears to imply that SOC influences oral health among Norwegian adults irrespective of financial and non-financial social characteristics. The present findings support the role of SOC as an independent covariate of various oral health outcomes, and are in line with previous national and international studies in the field [21, 27–29].

A strength of this study is the use of all items in the 13-item Norwegian SOC inventory, suggesting that this instrument measures a phenomenon identical to Antonovsky’s original SOC construct [1]. Numerous previous studies have used various abbreviated versions of the SOC scale based on population- and convenience-based samples [11, 12, 27]. Use of a large population-based sample covering a wide age range (16–79 years) of Norwegian adults as well as use of advanced statistical methods are further strengths of this study. The choice of CFA in contrast to explorative factor analysis (EFA) enabled the testing of a theoretically predefined factor structure and its equivalence across subgroups. Following Byrne [24] and Brown [25], legitimate comparison of structural relations across groups requires equivalence of the underlying measurement model. Testing the modified 1-factor model across genders simultaneously, confirmed equivalence of the unidimensional factor structure and factor loadings. This fulfils the requirements for an invariant measure that both the number of factors and the patterns of loadings must correspond in addition to equivalent factor loadings [24]. The present findings argue for absence of differences between men and women in the Norwegian adult population regarding their responses to the 13-item SOC scale.

The findings of this study should be interpreted in light of a number of limitations. First, the study design was cross-sectional and thus precluded any verification of temporal events and conclusions about causal relationships. Moreover, since the data were self-reported, there might be biases due to social desirability and the fact that respondents might be a selection of those who have particular interest in the research topic presented. Categorising the exposure and outcome variables into dichotomous and trichotomous variables might have restricted the variance in those measures. Although having a large population-based sample of Norwegian adults, the response rate in this survey was rather low and thus may have resulted in selection bias and limited external validity of the findings. Attrition analyses suggested, however, that the main reason for non-responses was due to the length and sensitivity of the questionnaire utilised. It seems unlikely, that such factors should have seriously biased observations regarding the factor structure of the SOC scale. Comparisons of the socio-demographic distribution of participants with the corresponding official Norwegian population statistics of 2018, confirmed that regarding certain socio-demographic characteristics the participants were representative of the respective Norwegian adult population.

The present findings accord with previous population-based studies regarding the unidimensional structure of an abbreviated SOC instrument as well as the robust association observed between strong SOC and a range of positive oral health outcomes in adults [11, 17, 29]. Based on a sample of Finnish adults, and using an abbreviated 12-item SOC scale, Bernabe et al. [5], achieved acceptable fit for a modified 1-factor model suggesting that the three components, comprehensibility, manageability and meaningfulness, should be merged when measuring SOC in the Finnish adult population. Moreover, based on a sample of the adult population in northern Norway, Holde et al. [17] provided support for a uni-dimensional SOC construct. In contrast, Feldt et al. [9] argued for a three-factor solution of the 13 item SOC scale in a Finnish working aged population. Accordingly, Mathisen et al. [27] argued for a three factor solution of an 11-item version of the Orientation to Life questionnaire when tested among Norwegian adults. The one-factor model supported in this study, however, accords with the argument by Antonovsky that the three SOC components should not be considered independent as the SOC scale was meant to measure one global orientation [1].

In conceptual models, socio-demographic characteristics and SOC are recognised as, respectively, distal and more proximal factors influencing oral health outcomes [30]. Accordingly, the present study revealed that SOC and a range of socio-demographic factors were independent covariates of various oral health outcomes [17, 29]. As demonstrated by the present study, participants with higher household income were less and more likely to express oral impacts and positive oral attitudes. However, some of those associations became insignificant when SOC was included in the final multivariable models, suggesting that SOC explains some of the socio-demographic variations in oral health. This makes SOC important in understanding how socio-demographics affects Norwegian adults’ oral health as reported by other studies [31–32]. Interestingly, the association between stronger SOC and less reported periodontal symptoms accords with another study using perceived periodontal health as the outcome variable [18] – but is at odds with those suggesting a positive relationship between stronger SOC and worse clinical periodontal status [17, 18].

This study demonstrated construct validity of the Norwegian version of SOC 13 and that in the presence of socio-demographic factors, SOC is an important contributor to oral health outcomes. Sense of coherence should be considered in oral health educational and promotional interventions among Norwegian adults.
